# E-cigarette Use in Prisons With Recently Established Smokefree Policies: A Qualitative Interview Study With People in Custody in Scotland

**DOI:** 10.1093/ntr/ntaa271

**Published:** 2020-12-26

**Authors:** Ashley Brown, Rachel O’Donnell, Douglas Eadie, Allison Ford, Danielle Mitchell, Alison Hackett, Helen Sweeting, Linda Bauld, Kate Hunt

**Affiliations:** 1 Institute for Social Marketing and Health, Faculty of Health Sciences and Sport, University of Stirling, Stirling, UK; 2 Faculty of Health Sciences and Sport, University of Stirling, Stirling, UK; 3 MRC/CSO Social and Public Health Sciences Unit, University of Glasgow, Berkeley Square, Glasgow, UK; 4 Usher Institute and SPECTRUM Consortium, College of Medicine and Veterinary Medicine, University of Edinburgh, Edinburgh, UK

## Abstract

**Introduction:**

E-cigarettes were one measure introduced to help people in custody (PiC) to prepare for and cope with implementation of comprehensive smokefree policies in Scottish prisons. Our earlier study explored experiences of vaping when e-cigarettes were first introduced and most participants were dual tobacco and e-cigarette users. Here we present findings of a subsequent study of vaping among a different sample of PiC when use of tobacco was prohibited in prison, and smokefree policy had become the norm.

**Methods:**

Twenty-eight qualitative interviews were conducted with PiC who were current or former users of e-cigarettes in prison, 6–10 months after implementation of a smokefree policy. Data were managed and analyzed using the framework approach.

**Results:**

PiC reported that vaping helped with mandated smoking abstinence. However, findings suggest that some PiC may be susceptible to heavy e-cigarette use potentially as a consequence of high nicotine dependence and situational factors such as e-cigarette product choice and availability in prisons; issues with nicotine delivery; prison regimes; and use of e-cigarettes for managing negative emotions. These factors may act as barriers to cutting down or stopping use of e-cigarettes by PiC who want to make changes due to dissatisfaction with vaping or lack of interest in continued use of nicotine, cost, and/or health concerns.

**Conclusions:**

E-cigarettes helped PiC to cope with smokefree rules, although concerns about e-cigarette efficacy, cost, and safety were raised. PiC may desire or benefit both from conventional smoking cessation programs and from interventions to support reduction, or cessation, of vaping.

**Implications:**

Findings highlight successes, challenges, and potential solutions in respect of use of e-cigarettes to cope with mandated smoking abstinence in populations with high smoking prevalence and heavy nicotine dependence. Experiences from prisons in Scotland may be of particular interest to health and/or justice services in other jurisdictions, with similar legislation on e-cigarettes to the United Kingdom, who are planning for institutional smokefree policies in their prisons or inpatient mental health settings in the future.

## Introduction

Smoking fulfills social, cultural, and psychological functions in prisons, such as maintaining personal or group identities, as a means of coping with boredom, environmental stressors, or poor mental health, and as a form of currency.^1–4^ Given this, and beliefs that prohibition of smoking reduces the already limited choices of people in custody (PiC) and so may be seen as a “punishment” and is likely to cause hardship and other difficulties in prisons,^5–7^ it is unsurprising that there is lower support for smokefree policies among PiC than among staff.^8^ However, it is important to note a substantial minority of PiC are more supportive of smokefree rules^8^ because of desires to quit smoking or perceptions that changes in smoking policy are beneficial for themselves and others.^7,8^ The availability of e-cigarettes in smokefree prisons has the potential to ameliorate possible challenges of no smoking rules for PiC who are unwilling or unable to stop use of nicotine, and may improve the health of a disadvantaged group if e-cigarettes are used for harm reduction in the longer term.

Given the well-recognized embedding of smoking in prison culture, removing tobacco from prisons is seen to be challenging. Yet, implementation of smokefree policy in prisons in Scotland and other jurisdictions^9–12^ is generally regarded as having been a success. There were no reported major incidents when the policy was implemented in Scotland.^13^ In addition, air quality measurements across Scottish prisons showed a drop of around 90% in secondhand smoke levels compared with measurements taken before the decision to implement the smokefree policy,^14^ confirming that it led to the virtual elimination of secondhand smoke,^15^ with expected health benefits for staff and PiC.

Prior to policy implementation on November 30, 2018, measures were introduced to support PiC to transition to smoking abstinence (starting from a 72% smoking prevalence^16^), including the expansion of existing smoking cessation services offering behavioral support and pharmacotherapy, extensive communication strategies, and strong health and justice partnership-working to support PiC to prepare for the change in smoking rules. In addition, rechargeable e-cigarettes became available in prisons for the first time (from September 2018). This was in line with contemporary expert consensus in the United Kingdom that e-cigarettes pose less risk to health than smoking tobacco, beliefs that use of e-cigarettes for harm reduction should *not* be discouraged by public policy or health professionals,^17–19^ and the fact that e-cigarettes are not covered by Scotland’s smokefree legislation. The policy on e-cigarette use among PiC in Scotland is consistent with those in place in prisons in some other jurisdictions (eg, England and Wales, Isle of Man, and some US states), and is (partially) aligned with rules in respect of e-cigarette use in other public places in Scotland and England, including those covering some National Health Service (NHS) premises.^20,21^

However, little is known about use of e-cigarettes in prisons with smokefree policies, thus undermining evaluation of their net benefits and risks at individual and prison population levels. We previously reported on initial views and experiences of e-cigarette use (vaping) among PiC in Scotland^22^ when e-cigarettes were first allowed shortly *before* implementation of the smokefree policy. At that time, e-cigarettes were novel (single-use e-cigarettes first became available for use by PiC in designated spaces in prisons from ~February 2018 and rechargeable e-cigarettes from September 2018), and PiC had very limited choice of e-cigarette products compared with the general population. Participants in our earlier study, most of whom were dual tobacco and e-cigarette users, expressed strong support for the introduction of rechargeable e-cigarettes (hereafter “e-cigarettes” unless otherwise stated) in prison, and voiced expectations that e-cigarettes would help PiC, a population with high nicotine dependence, to cope with future mandated smoking abstinence. At the time of that study, most participants’ vaping behaviors were not firmly established. Even so, some important issues with symptom relief were raised, and some expressed surprise or discomfort about the frequency/amount that they (or others) were vaping. Perceptions of benefits of switching from smoking to vaping in prison varied; however, it is notable that some participants were cautiously optimistic, especially in relation to cost, on the basis of initial experiences.

Here we present findings of a subsequent study. Our aim was to extend previous evidence by exploring views and experiences of e-cigarettes among PiC, in prisons in a jurisdiction with well-established smokefree policies (ie, when smokefree rules and vaping behaviors had had a chance to fully embed and PiC could no longer dual-use e-cigarettes and tobacco). Like our previous study, it was conducted in Scottish prisons, but with a *different* sample of PiC.

## Methods

Qualitative interviews were conducted with PiC who were current or former users of e-cigarettes in prison, 6–10 months after legislative changes had prohibited smoking in Scottish prisons from November 30, 2018. Ethical approval was granted by the SPS Research Access and Ethics Committee and University of Stirling General University Ethics Panel (GUEP 497).

### Sampling and Recruitment

Methods for this study were almost identical to those reported in detail for our earlier vaping study,^22^ conducted immediately *before* smokefree policy implementation. In both, interviews were conducted within six Scottish prisons selected to include diverse populations (by age, sex, and sentence length). The research team provided a designated staff contact (in a managerial role) in each prison with guidance on the desired sample size and characteristics. Potential participants were first approached about the study by these staff, who arranged a meeting with a research team member for those who expressed interest in participating. Taking account of the literacy and learning needs of PiC, researchers provided PiC with written and verbal information about the study and checked that participants felt informed about the study and willing to consent (those who agreed provided consent either verbally or in writing on a case by case basis) before the interview proceeded. Of the 28 participants (see Table 1) included in this analysis most were as follows: convicted (*n* = 21, 11 serving ≤4 years; nine serving over 4 years; one did not report sentence length); men (*n* = 20); and aged ≤40 (*n* = 16, two did not report their age).

**Table 1.** Participant Characteristics

Vaping habit	
Daily	23
Weekly or more	1
Not at all	3
Missing	1
Whether the participant had tried e-cigarettes prior to prison?	
Yes	18
No	7
Missing	3
Sex	
Male	20
Female	8
Age	
18–30	11
31–40	5
41–50	7
51–60	2
≥61	1
Missing	2
Remand/convicted status	
Remanded	7
Convicted	21
Sentence length	
3–12 mo	3
1–4 y	8
≥5 y	9
Missing	1
How long has the participant been in prison on this sentence (not asked of people on remand)	
Up to 90 d	3
3–12 mo	7
1–4 y	8
≥5 y	2
Missing	1

### Data Collection

In-depth interviews (average ~43 minutes) were conducted in person in May–August 2019 (by AB, RO, KH, DE, and AF) using a topic guide covering: participant background; smoking and vaping history; experiences and perspectives of vaping in prison; and views on benefits/risks of e-cigarettes being available to PiC. Researchers could vary the question wording, topic order, and use of probes and prompts to gain additional detail or stimulate discussion. Participants were invited to raise any additional points they thought were important. In line with prison service research guidance in Scotland, no financial incentives were offered for participation.

For context, interviews were conducted prior to (Autumn 2019) media reports that discussed vaping and cases of lung injury in the United States,^23^ and over a year before the first documented Covid-19 cases.

### Analysis

With written/audio-recorded permission, interviews were recorded and transcribed. De-identified transcripts were thematically analyzed using the framework approach, in line with the principles and methodological underpinnings described by Spencer et al.^24^ AB led on producing a thematic framework based on reading transcripts in light of the study questions, topic guide, and relevant literature. Transcripts were summarized into the framework grid (row = participant, column = theme) in NVivo 12 by AB, RO, DM, and AH, to synthesize content before detailed analysis. This involved writing summaries of the data in the relevant cells in the framework grid and creating hyperlinks to transcripts to support data retrieval during the analysis process. AB reviewed all summaries to check consistency and interpretations. AB and RO identified high-level themes from the summaries and data excerpts. AB then conducted a more granular analysis of the data to understand the range of experiences and perspectives on vaping, examine patterns, and interpret meaning. Themes are presented below alongside selected excerpts from interviews, which indicate participant characteristics: prison/serial number (randomly selected for this paper to preserve anonymity), custodial (remand [R], short-term conviction [ST], long-term conviction [LT]), and vaping status (current vaper [CV], ex-vaper [ExV]).

### Context

The study context is described in more detail elsewhere,^22^ but we summarize salient points here. Under the smokefree rules, PiC are prohibited from smoking tobacco in all areas of prison but can use a limited selection of e-cigarette products, stocked in the prison shop (“canteen”), in their room (cell) and selected outdoor areas; staff are not permitted to use e-cigarettes on prison property. The two brands of rechargeable e-cigarettes on the canteen list at the time of the interviews were closed tank systems taking prefilled e-liquids (highest strength = 18 mg/mL). The higher upfront costs for one brand are noteworthy given PiC’s restricted incomes (for some, their only income source is their prison wage which can range from ~£5 to £21^25^ and limits on weekly expenditure are implemented). The strongest e-liquid (18 mg/mL) for the cheaper device was only available in tobacco flavor. An e-cigarette product list for prisons in Scotland is available elsewhere.^26^

At the time of the interviews, Prison “Quit Your Way” services^27^ were committed to providing free behavioral support to PiC opting to “withdraw[al] from nicotine using e-cigarettes.” Specific guidance^26^ to support PiC to stop (or cut down) vaping has been published subsequently, informed by the preliminary results of this study.

## Results

### Background

#### Participants

All participants were tobacco smokers before either the smokefree policy implementation or their current imprisonment (if that commenced after November 30, 2018). Similarly to our earlier study,^22^ many found it challenging to imagine whether or not they would return to smoking on release from prison; however, very few expressed no or little interest in remaining abstinent. Twenty-five participants were currently using e-cigarettes (almost all daily) and the other three had tried e-cigarettes in prison but no longer vaped. In common with other vapers,^28^ participants found it difficult to characterize their vaping habits in terms of number of vaping sessions per day or the number of puffs per session. Vaping behaviors varied between individuals and over time (see below).

#### Views on Making E-cigarettes Available in Smokefree Prisons

Consistent with expectations expressed by participants in our earlier study, PiC in this sample generally perceived e-cigarettes to have been an important part of the process of removing tobacco from Scottish prisons without major disruption, by providing an alternative to smoking. They also suggested that e-cigarette availability contributed to the continuing successful implementation of smokefree rules now the policy had had at least 6 months to embed.

B2-LT-ExV: for the smoking ban, the smartest thing they did was put vapes in here.

### Vaping Behaviors in Prison

Several notable features of vaping among PiC were identified. First, e-cigarettes no longer had the “novelty” status in prisons that was so salient in our earlier study. Second, vaping tended to be a habitual activity; almost all current vapers vaped daily, thus potentially developing entrenched behavior which might be difficult to change. Third, e-cigarettes were used, sometimes alongside nicotine replacement therapy, both for coping with mandated smoking abstinence, and in response to physical or psychological factors (eg, nicotine cravings, to mimic previous smoking rituals or behaviors, curiosity, seeking pleasure, feeling bored, stressed, or angry). An additional reason given by some participants for using e-cigarettes was to support a move to long-term smoking cessation, including possibly vaping postliberation as an alternative to smoking.

Fourth, some participants’ accounts suggest several potentially context-specific practices of vaping had emerged among PiC by the time of the current study. There were striking descriptions of heavy or excess vaping by themselves and/or others. Some participants expressed surprise or concern about their own vaping, whereas others spoke about it as a matter of fact or as something they were resigned to. For example, there were descriptions of holding the device for extended periods while in their rooms (cells) (“*if it’s out of my hand people are surprised, and it is constant*” C1-LT-CV); taking many and/or heavy draws during vaping sessions (“*I’m just constantly pressing it and just keeping…inhaling, inhaling, inhaling*” M1-R-CV); and consuming what participants perceived as a relatively high number of e-liquids per week and running out of supplies between deliveries from the “canteen.” Some participants’ accounts of vaping might be described as a form of “binging” behavior, potentially indicating some challenges with self-regulation of nicotine intake when using e-cigarettes and possibly resulting in short-term effects from too much nicotine.

L2-LT-CV: When you’re vaping, …you don’t even notice it [nicotine], until I’ve been sitting going at it constantly for 10, 15 minutes, and I start to feel a little bit sick, and then I think, that’s probably nicotine overdose right there.

Other vaping practices were also described; these participants reported more moderate use of e-cigarettes in prison in terms of regulating e-liquid intake, and vaping behavior. Vaping practices in prison often varied over weekdays/weekends, and over the course of someone’s imprisonment, with examples of moving from heavier to more moderate vaping and vice versa.

PiC’s vaping practices (including frequency of vaping and number of e-liquids refills consumed) were potentially influenced by a combination of individual-level and situational factors. Individual-level factors included someone’s level of nicotine dependence, the degree to which they found vaping pleasurable, and the extent to which they had become habituated to vaping. Flavored e-liquids continued to be part of vaping’s appeal for many participants, as did aspects of vaping which replicated (eg, hand movements and inhaling/exhaling vapor) or improved (eg, smell and taste) elements of smoking. In relation to situational factors, extended periods of time in their rooms with limited distractions or alternative activities, particularly over the weekend, provided significant opportunity and perceived reasons to vape, particularly since vaping was described as an easy and enjoyable habit and a strategy for managing negative emotions in prison, especially boredom.

L1-R-CV: you end up over [vaping]…you’re in your cell, you’re bored, you don’t know what else to do, and you just start puffing. And you end up getting to the point like that, I’m going to end up with nothing [no e-liquids].

Heavy or “binging” vaping practices were also potentially facilitated by difficulties some were experiencing in managing nicotine dependence with the products available in prison. Some participants (eg, B5-ST-CV) expressed frustration that the e-cigarettes sold were not “*strong enough*.” Others were not using the more powerful of the two devices available for reasons of cost or personal preference, or were reluctant to use the strongest (18 mg/mL) e-liquid sold for the cheaper device because it was only sold in a flavor (tobacco) they did not like. Thus, the e-liquids used were partly determined by individual preference in respect of flavor and nicotine strength and partly by practical considerations in terms of what e-liquids could be purchased or borrowed in prison. For some PiC, e-liquid flavor was a greater driver of product choice than nicotine strength in a context in which a very limited range of products are available to purchase.

### Experiences and Perspectives on Vaping in Prison

#### Experiences of Vaping

As in our earlier study, disposable e-cigarettes were unpopular except as an interim measure for new admissions, or when purchased occasionally to mimic certain aspects of smoking. With respect to rechargeable e-cigarettes, some participants had adapted relatively quickly to a different system of nicotine delivery and were finding vaping to be a reasonably functional (M3-ST-CV: “*does the job*”) or satisfying activity, in light of mandated smoking abstinence. However, some still “missed” certain aspects of smoking, for example, nicotine buzz or the act of rolling tobacco.

Others indicated negative experiences, and frustrations, with vaping in prison. This partly reflected significant ongoing difficulties relieving symptoms, even if e-cigarettes were regarded as “better than nothing” in the circumstances. For some it also resulted from limited motivation to ever quit smoking and perceptions that vaping was not something they were doing in prison by choice (“*(vaping) was…what’s the word? It was forced on us*” O3-ST-CV). In contrast, for others it reflected strong aspirations to end nicotine dependency and worries about whether they would manage to quit vaping in the future.

A more prominent issue in this study compared with our earlier study was that some e-cigarette products were reportedly faulty/lacked durability, leaked, or tasted burnt. These problems appeared to result from erroneous product use, incorrect handling of e-liquids, poor maintenance of devices, or repurposing of e-cigarettes for use of new psychoactive substances (which may have adverse consequences for safety in prisons^29^):

C6-ST-CV: …all of a sudden it [e-cigarette] stops working…you’re not supposed to tamper with them, but people do try and mess about and find out what’s going on…they’ve also been used for taking drugs…

Some voiced strong comments about the need for a greater range of e-cigarette products in the canteen, including more powerful devices, and greater variety of e-liquids strengths/flavors.

#### Health Benefits and Risks of Vaping

Our previous study noted uncertainty, and some concern, among participants about the safety of e-cigarettes, partly because of the absence of long-term studies. This was also clear in the current study, with some strong statements that this generation of vapers was like “*guinea pigs*” (L3-LT-CV). It was suggested that PiC were distinct from other vapers, as the only group in society who were mandated to abstain from smoking at all times.

C2-ST-CV: …I’ve got…split minds about it [e-cigarettes in prison]…It’s the long-term things that bother me, I don’t want to be like one of those…people that ended up dying because of the asbestos…I don’t want to be like mice being tested on…But there are hundreds of people doing it outside as well, so I don’t know.

Perceptions of the balance of health risks and benefits of vaping also continued to vary. These ranged from participants stating that vaping is “*like switching your cola for diet [cola]*” (B1-ST-CV), to others suggesting that e-cigarettes may cause similar or greater harm than tobacco. Views on the absolute/relative safety of vaping were potentially (mis)informed by a range of factors, including some that were specific to prisons. Perceptions of perceived short-term health effects of switching to vaping potentially influenced views on the safety of e-cigarettes more generally. While some reported perceived improvements to their health (eg, improved sense of taste, reduced respiratory problems) after switching to vaping in prisons, a few attributed some more acute health problems (eg, bleeding gums, chest pains, lung problems) to vaping. General concerns were raised about the potential for vaping to contribute to lung damage/disease and about transmission of illness through sharing e-cigarettes:

M4-Missing-CV: People will go into other people’s rooms to get a puff of their vape…everybody is [doing it]. I’ve got a viral infection; I think it might have been caught [from] one of the vapes.

Other factors potentially influencing perceptions of safety included media and word of mouth stories about vaping, including accounts of supposed links between vaping and “popcorn lung”: …*there’s something about them [e-cigarettes] I don’t trust…[…]*…*there’s a boy [PiC]…went away down south [to an English prison]…And he…says that down there, the other inmates are suffering from popcorn lung…* (O2-LT-ExV).

Regarding prison-specific factors, prison policy on e-cigarettes was potentially informing perceptions of “safety.” There was a suggestion that the distribution/sale of e-cigarettes in prison (and indeed wider society) were indications of product “safety”: “…*obviously, it’s not [bad for you], or obviously they wouldn’t be getting sold*” (C4-R-CV). Hence, some participants questioned the logic of restricting vaping indoors in prisons on health grounds, or found the rules confusing:

M6-ST-CV: if they’re [e-cigarettes] not harming anybody, I don’t see why we can’t smoke [vape] them out in the hall…?

#### Cost and Access

Participants’ expenditure on e-cigarettes varied, reflecting use patterns, product choices, and individual financial resources. Some remarked on the potential positive financial implications of switching from smoking to vaping in prison, such as being able to use cost savings to buy healthier items from the canteen or afford increased family phone contact:

G2-ST-CV): I don’t run out of [phone] credit now…It’s definitely a plus…giving up smoking, definitely…you’re…saving a lot of money [by switching to vaping].

Nonetheless, a prominent theme was concern about the affordability/value for money of vaping in prison. These perceptions potentially stemmed from some believing that vaping was a less pleasurable alternative to smoking and was driven by circumstances rather than by choice; perceptions that closed-system devices were not good value for money; and concerns about the relatively high price of products, for example, for PiC with very low incomes (who previously had had the option of low-cost pipe tobacco prior to the smokefree policy):

L6-LT-CV: If you’re living on a [basic] wage…You could buy one packet…[of e-liquids per week]. That leaves you, what, two pounds to get your coffee, teabags, sugar, phone calls…Toiletries…it would be better…if the refills were bigger or… you could buy the wee … bottles you fill up yourself….

Specific challenges were discussed in relation to meeting the upfront costs of vaping in prison (eg, because of initial delays in accessing money for new admissions), the cost of replacing devices or chargers, and balancing spending on e-liquids and other canteen items/savings. These cost factors potentially contributed to difficulties which some were experiencing in managing nicotine dependence in prison after the smokefree policy.

Similar to tobacco products pre-smokefree rules, participants described practices of mutual support, which had developed in relation to accessing e-cigarette products. Examples included someone leaving their device for a friend when they were released, and letting others take a few draws from their vape or loaning them an e-liquid if they ran out. However, it was noted that these arrangements could lead to irritation or tensions (“*You’re constantly fighting over a vape*” B3-ST-CV) among PiC, although the data suggest problems were not dissimilar to those previously caused by tobacco.

Additionally, it was reported that e-cigarettes, like tobacco pre-smokefree rules, had become part of the (unofficial) prison economy, creating another source of conflict and debt. As the quote below illustrates, prefilled e-liquids were not easily divisible into small quantities and so were less convenient than tobacco to share/trade:

B4-ST-CV: But people can get into a lot of debt and then they can come to the next canteen and you’ll not have enough money to buy the…oils [e-liquids].

**I: That used to happen with tobacco – people got themselves into tobacco debt?**

B4-ST-CV: Tobacco was a bit easier though to deal with because you could just rip a bit off the tobacco and give somebody that and they’d be happy, but you can’t give them half a [e-liquid].

Some expressed a strong desire for the prison service to explore options for easing the financial burden of vaping on PiC, and for improving prompt access to e-cigarettes (or alternative nicotine products, depending on individual preference) for new arrivals.

### Future E-cigarette Use

Current vapers differed in their ideas about whether or not they might continue vaping, both within prison and after release, and many expressed ambivalence or uncertainty about what the future in general might hold. Key reasons for potentially continuing to vape in future included finding vaping enjoyable or believing it had benefits as an alternative to smoking or as a smoking cessation aid while in prison and potentially after release:

O1-LT-CV: yeah [I’ll continue to vape in prison], because there’s no other alternative. Like, if you can’t smoke tobacco then you’ll use the vapes because it’s the next best thing.

L5-R-CV: using it in the prison has made me think what it’s like to use it…I’ve thought, well, maybe at first when I get out, I can mix the two, and then gradually go down to just using the vape.

However, reasons for potentially cutting down or quitting vaping in future, or for having already done so in prison, were also provided. These included negative experiences of vaping; worries about “safety” (“*it’s not as bad as smoking. But you’ve still got health issues that come with it*” C5-R-CV) or cost (“*[if] you save…£30 a week it’s a lot of money*.” C3-LT-ExV); wanting to end any dependence on nicotine; not wanting to replace one “habit” with another; and changes in circumstances which reduced/eliminated the need to vape (eg, distractions or access to tobacco postrelease).

Some expressed concerns that making e-cigarettes available in prison might be detrimental to long-term smoking or nicotine cessation, and some voiced regret about their own uptake of vaping in prison. For instance, the quote below illustrates the role of situational and psychological factors in prisons in driving uptake of vaping by a participant who would like to move beyond, rather than feel “trapped” (C1-LT-CV) by, nicotine dependence.

C2-ST-CV: I would…rather…have stopped smoking [withdrawn from nicotine] when I came in…I wasn’t bothered about that vape until about three weeks into my sentence…taking my medication away, putting my stress up [were reasons for vaping] definitely.

PiC who want to reduce or stop vaping may benefit from support to achieve their goals; e-cigarettes were viewed by some as habit-forming and potential situational and psychological barriers to making behavior change in prison were mentioned. Suggestions for measures included, incorporating e-cigarette users into existing “Quit Your Way Prison” services, including offering nicotine replacement therapy, and for options to purchase lower strength e-liquids to reduce nicotine intake.

C6-ST-CV: I don’t think it’s going to be easy [to quit vaping]…in here you know you’ve got to drop from 18mgl (e-liquids) to 12mgl and then from 12gml to nothing, at the moment. It’s not feasible for people to gradually reduce nicotine intake with e-liquids…just now.

## Discussion

To our knowledge, this study is the first to internationally explore e-cigarette use across several prisons in a prison system that has become smokefree. Using rigorous methods for data collection and analysis, it provides new insights on e-cigarette use, presenting novel qualitative data collected from PiC in Scotland 6–10 months after smokefree policy was successfully introduced. It extends and complements our previous study of vaping,^22^ which involved interviews with a different sample of PiC, conducted in a very specific context, that is, very soon after rechargeable e-cigarettes were introduced, when tobacco was still permitted and very few were using e-cigarettes exclusively. Findings from the current study suggest that vaping in Scottish prisons continued to be strongly influenced by circumstances (ie, smokefree rules) and a desire to fulfill needs previously met by smoking. Consistent with expectations expressed by PiC in our earlier study, vaping was seen as an important tool to help them manage under smokefree rules and was replicating some of the psychological needs (eg, countering boredom) and cultural functions of smoking, as is the case for many ex-smokers in the general population who vape.^28^ Many of the factors influencing vaping behavior in prisons mirror those for smoking, although participants’ vaping habits did not necessarily replicate their previous smoking habits. For those PiC not wishing to stop nicotine use, e-cigarettes may potentially support long-term tobacco harm reduction, but further studies are required to test this hypothesis.

In contrast to our earlier study, there were more striking descriptions of heavy or excessive use of e-cigarettes amongst PiC. This type of use may be driven by “compensatory” behaviors in a population with high levels of nicotine dependence^30^ and no access to tobacco, and by factors such as enjoyment, use of e-cigarettes for emotional regulation, and boredom. There is evidence of compensatory behavior (eg, puff numbers and duration) from general population studies of vaping under high/low nicotine conditions.^31^ It is worth noting that the products available in UK prisons complied with the EU limit of less than 20 mg/mL nicotine content.^32,33^ This may be insufficient for some heavy smokers and result in more frequent or intensive vaping (the “compensatory” behavior mentioned above) to avoid symptoms of nicotine withdrawal. However, it is unclear from evidence published to date whether heavy/“compensatory” vaping has been observed in other groups under institutional smokefree policies, for example, mental health service users, including in the United Kingdom , where nicotine limits for vaping products apply.

Heavy/“compensatory” use of e-cigarettes is likely to be of some concern in justice and health services, as it may place strain on the finances of people relying on very low incomes, lead to feelings of frustration, dissatisfaction, and discomfort if it does not provide adequate craving relief,^31^ or be contrary to an individual’s goals for nicotine reduction or cessation. The implications for physical health are unclear. Questions remain about potential links between heavy vaping of (low) nicotine concentration e-liquids and higher exposures to formaldehyde,^31^ and the effects of continued vaping at any level are not yet fully known.

Consistent with other user groups,^34–36^ poor experiences with vaping, health concerns, cost, and not wanting to continue to use nicotine were reasons for wanting to reduce or stop vaping. It will be important in future studies to explore whether PiC go on to develop higher or lower tolerance levels for (dis)continuing vaping compared with other groups of vapers given some distinct push–pull factors in prisons, for example, absence of tobacco, limited e-cigarette product availability, and some specific vaping norms and practices among PiC. We are unable to compare our results to other studies of e-cigarette use in prisons in other jurisdictions, since none, to our knowledge, have been published.

Overall, the findings suggest that making e-cigarettes available in prisons in Scotland has supported individual smokers and the prison service to successfully undergo a potentially very challenging process of change. This enabled smokefree rules to become the new norm in prisons, protecting nonsmokers from secondhand smoke exposures. Yet the findings also signal a need for future studies of e-cigarette use in prisons. In particular to determine the balance of potential gains if e-cigarettes are adopted long-term for harm reduction against any negative effects if e-cigarette use principally becomes a new form of (potentially unwanted and costly) dependence in prison. Although many PiC may find e-cigarettes more acceptable than conventional smoking cessation treatments in smokefree prisons, some feel strong dissatisfaction about not having greater control over their behavior and/or have concerns about use of a novel and habit-forming product in prison. It will also be important for ongoing monitoring to understand the needs and experiences of these groups. In the absence of evidence on the longer-term implications of e-cigarette use in prisons, other jurisdictions opting to permit vaping among PiC may wish to consider implementing measures to minimize any unintended negative consequences, as has been the case in Scotland (see below). Other jurisdictions could consider making e-cigarettes available in prisons on an interim basis only, to reduce potential problems. However, such a policy may seem to be at odds with understandings of smoking dependence as a chronic condition^37^ and would erode the choices of PiC.

The study is novel and has utilized robust, well-established methods for sampling, collecting, analyzing, and reporting qualitative data for applied policy research. We believe that insights are transferable to prison systems in countries with similar regulations on e-cigarettes to the United Kingdom and potentially to other smokefree settings supporting heavy smoking groups. Ongoing work is exploring the views and experiences of prison staff about the use of e-cigarettes by PiC, including challenges that e-cigarettes present in this context, and analyzing “canteen” spend on nicotine-related (and other) products in the lead up to and following implementation of the smokefree policy to explore whether (and how) levels of spending on nicotine products among PiC has changed over time.

The study also has some limitations. Although our sample represents a diverse range of PiC (with respect to sex, age, and sentence length) from six prisons, it is possible that some vaping behaviors and experiences are not reflected because of (the essential) use of gatekeepers for recruitment or self-selection bias. Furthermore, constraints on interview length affected the amount of information that could be collected on participants’ smoking and e-cigarette use histories and it was not possible to triangulate interviews with other methods of characterizing vaping (eg, participant observation) to provide a fuller picture of vaping practices in prisons, and to aid comparison with other user groups.

The findings have several implications for prison policy and potentially for other smokefree settings, such as mental health inpatient settings. The introduction of e-cigarettes into Scottish prisons, which has been widely welcomed by PiC, may have discouraged some from using smokefree policy as an opportunity to become nicotine-free in the long term. It is thus essential that PiC have equitable access to conventional smoking abstinence/cessation aids such as nicotine replacement therapy, alongside e-cigarettes, particularly as they initially enter prison.

The study highlights several possible barriers to vaping reduction/cessation for PiC in smokefree prisons seeking to change behavior for personal preference, cost, or health reasons. In response to our findings, and feedback to the NHS from PiC and staff, Scotland has led the way in developing novel guidance^26^ to support advisors to meet the needs of PiC who want to reduce or stop vaping. Similar guidance is likely to be helpful in other jurisdictions where e-cigarettes are sold in smokefree prisons. We plan to evaluate the implementation and potential effectiveness of this guidance in a future study, to inform delivery, and to identify transferrable insights for other settings.

To improve the chances of success in cutting down or quitting vaping and improve overall health, the data suggest that greater access to activities that PiC find meaningful (eg, in-cell hobbies, prison gyms, education, or training) would be a useful adjunct to services supporting PiC in the management of nicotine dependence, particularly in reducing cultural and psychological drivers of vaping. In addition, PiC may benefit from information campaigns to discourage misuse of e-cigarettes, particularly in the light of concerns about use of psychoactive substances in prisons, and to support them to make more fully informed choices about e-cigarette use.^29^ Such campaigns could be delivered through short videos, for example, shown at prison induction, and one-to-one or in small groups by peer mentors and healthcare professionals. Consideration could also be given to further review of the range of e-cigarette products available, including potentially piloting new device(s), and identifying the optimal combinations of strengths and flavors of e-liquids for this user group. It will be important to consider growing evidence on risks/benefits of using higher or lower strength e-liquids and on whether e-liquid flavors support or hinder harm reduction when selecting products. It is likely that some distinct policies on vaping in Scottish prisons will emerge, to balance imperatives to support PiC, as appropriate, to minimize continued use of nicotine in a smokefree environment, and to reduce rates of relapse to smoking postrelease.

In conclusion, PiC report that vaping helped them cope with mandated smoking abstinence, although concerns about e-cigarette efficacy, cost, and safety were raised. Findings suggest that PiC could be susceptible to heavy vaping, particularly with current products available in the United Kingdom. PiC may desire or benefit both from conventional smoking cessation programs and from interventions to support reduction or cessation of vaping, as appropriate.

## Supplementary Material

A Contributorship Form detailing each author’s specific involvement with this content, as well as any supplementary data, are available online at https://academic.oup.com/ntr.

ntaa271_suppl_Supplementary_Taxonomy_Form
